# Price Transparency Compliance Among Hospitals Caring for Disadvantaged Populations

**DOI:** 10.1001/jamanetworkopen.2026.6312

**Published:** 2026-04-10

**Authors:** Daphne Hao, Vinay K. Rathi, Joseph S. Ross, Rosh K. V. Sethi, Roy Xiao

**Affiliations:** 1 Pace Center for Civic Engagement, Princeton University, Princeton, New Jersey; 2 Department of Otolaryngology–Head and Neck Surgery, The Ohio State University School of Medicine, Columbus; 3 Section of General Medicine, Department of Internal Medicine, Yale University School of Medicine, New Haven, Connecticut; 4 The National Clinician Scholars Program, Department of Internal Medicine, Yale University School of Medicine, New Haven, Connecticut; 5 Department of Health Policy and Management, Yale University School of Public Health, New Haven, Connecticut; 6 Center for Outcomes Research and Evaluation, Yale New Haven Health, New Haven, Connecticut; 7 Department of Otolaryngology–Head and Neck Surgery, Harvard Medical School, Boston, Massachusetts; 8 Division of Otolaryngology–Head and Neck Surgery, Brigham and Women’s Hospital, Boston, Massachusetts; 9 Center for Head and Neck Oncology, Dana-Farber Cancer Institute, Boston, Massachusetts; 10 Center for Surgery and Public Health, Brigham and Women’s Hospital, Boston, Massachusetts; 11 Department of Otorhinolaryngology–Head and Neck Surgery, University of Pennsylvania, Philadelphia

## Abstract

This cross-sectional study investigates rates of hospital compliance with payer-negotiated price transparency by extent of care for disadvantaged populations.

## Introduction

The Centers for Medicare & Medicaid Services (CMS) implemented the Hospital Price Transparency rule in 2021,^1^ requiring hospitals to publicly disclose payer-negotiated prices or face maximum fines of $2 million per year.^2^ While hospital price transparency has increased, compliance remains uneven^3^ and may be worse among hospitals with fewer resources, particularly those caring for more disadvantaged patients. These hospitals may be underresourced because of their patient populations or because they bring in less revenue for the same services. We used hospital- and payer-disclosed pricing data to examine compliance and pricing, focusing on 10 top oncologic surgical procedures because improved transparency may be associated with reduced financial hardship for patients receiving cancer care.

## Methods

This cross-sectional study used publicly available, nonpatient data and was therefore exempt from institutional review board (IRB) review by Mass General Brigham IRB. It is reported in accordance with the STROBE reporting guideline. We included hospitals participating in the CMS Inpatient Prospective Payment System (IPPS) with available pricing data from Turquoise Health to determine overall compliance (yes or no), defined as price information being mostly or completely available as usable, machine-readable files (eMethods in Supplement 1).

We characterized hospitals’ extent of care for disadvantaged patients using 2 metrics. First, we used the 2023 Lown Hospitals Index inclusivity score ratings, focused on racial and income inclusivity and categorized into 3 tiers: low (1-2), medium (3), or high (4-5).^4^ Second, we used CMS Disproportionate Share Hospital (DSH) patient percentage value from the fiscal year 2024 IPPS, categorized into tertiles (<22%, 22%-33%, and >33%).^5^

We compared hospital quarterly price compliance from quarter 1 (Q1) 2022 through Q3 2023 by hospital inclusivity and DSH percentage using discrete-time hazards modeling. We identified payer-reported negotiated prices for 10 top oncologic procedures to compare compliant and noncompliant hospitals (required by CMS to be reported by payers since July 1, 2022). Prices were adjusted for hospital wage index, summarized as a median per procedure at each hospital, and compared between compliant and noncompliant hospitals using student *t* tests. We selected an α level of .05 for statistical significance; Benjamini-Hochberg adjustment for 16 tests reduced the significance threshold to .0375. Two-sided tests were performed using R version 4.5.1 (R Project for Statistical Computing). Data were analyzed from January 2022 through September 2023.

## Results

Among 2464 hospitals (Table; eFigure in Supplement 1), those more inclusive of disadvantaged populations had lower compliance rates and were less likely to become compliant over time (Figure, A-C). For example, hospitals with low Lown race inclusivity had a higher hazard of becoming compliant over time (hazard ratio, 1.25; 95% CI, 1.05-1.48; *P* = .009).

**Table.** Hospital Characteristics

Characteristic	Hospitals, No. (%) (N = 2464)
Part of multihospital system	
Yes	2226 (90.3)
No	238 (9.7)
Ownership type	
Nonprofit	1633 (66.3)
For profit	483 (19.6)
Government	348 (14.1)
Beds, No.	
1-100	732 (29.7)
101-250	924 (37.5)
≥251	808 (32.8)
Teaching hospital	
Yes	1117 (45.3)
No	1349 (54.7)
Lown race index rating, No. stars^a^	
1	171 (6.9)
2	238 (9.7)
3	1737 (70.5)
4	232 (9.4)
5	86 (3.5)
Lown income index rating, No. stars^a^	
1	133 (5.4)
2	432 (17.5)
3	1243 (50.4)
4	462 (18.8)
5	194 (7.9)
Location	
Urban	1426 (57.9)
Rural	1040 (42.2)
Region	
Northeast	379 (15.4)
Midwest	566 (23.0)
South	1037 (42.1)
West	482 (19.6)
Wage index, mean (SE)	1.034 (0.004)
Mean daily census, mean (SE)	150 (4)
DSH, mean (SE), %^b^	0.314 (0.003)

Abbreviations: DSH, Disproportionate Share Hospital; SE, standard error.

^a^ Lown indices are graded on a scale of 1 to 5 representing increasing levels of inclusivity.

^b^ The DSH percentage represents patient percentage values from the Centers for Medicare & Medicaid Services fiscal year 2024 Inpatient Prospective Payment System file.

**Figure.**
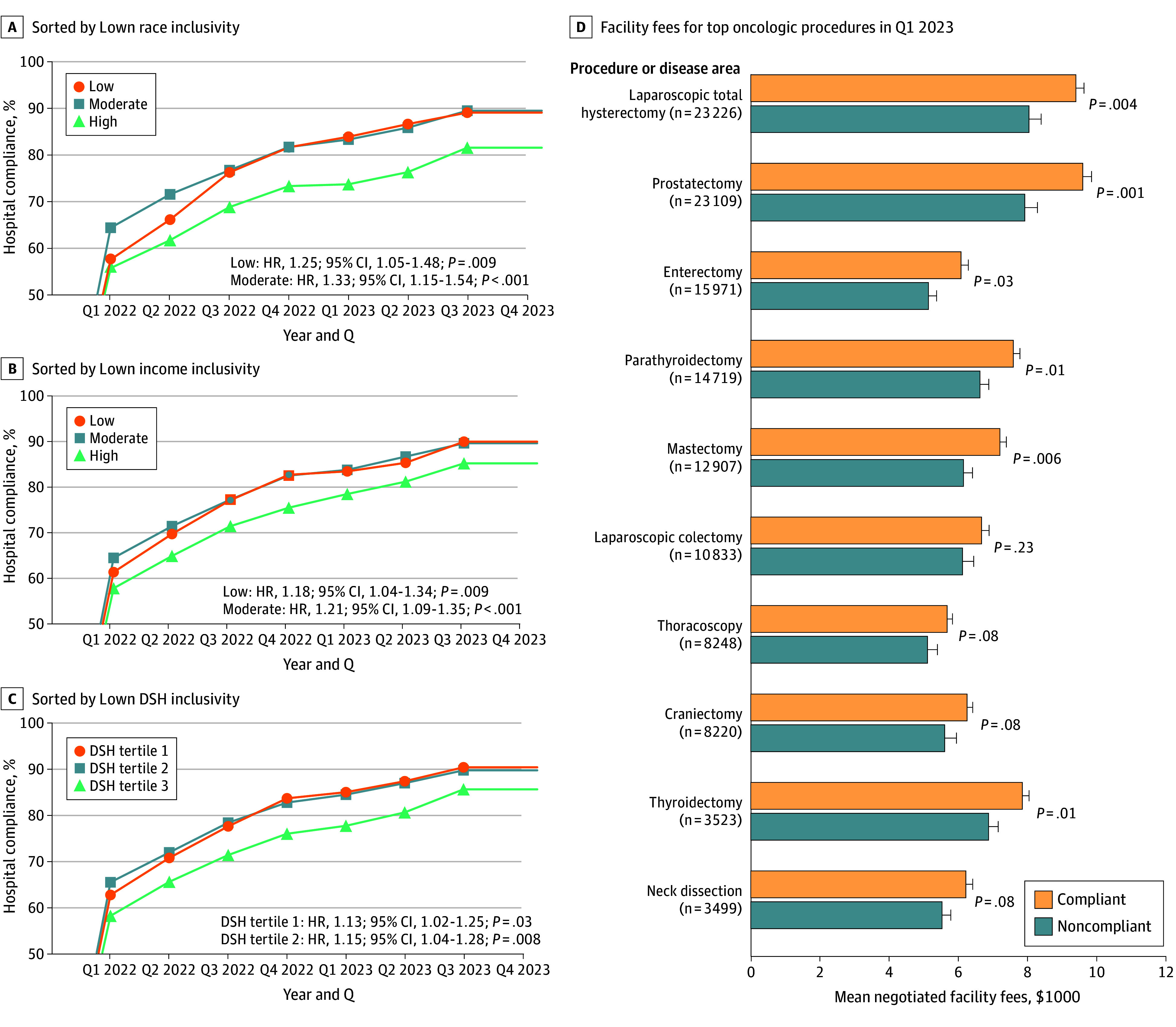
Hospital Compliance Over Time and Negotiated Facility Fees A-C, Hazard ratios (HRs) are calculated and reported with 95% CIs using the high-inclusivity group as the reference group. D, Mean facility fees for the top oncologic surgical procedures in quarter 1 (Q1) 2023 are presented. DSH indicates Disproportionate Share Hospital; error bars, standard errors.

As of Q1 2023, compliant hospitals had significantly higher mean negotiated facility fees for most oncologic procedures (6 of 10 surgery types [60.0%]) compared with noncompliant hospitals (Figure, D). For example, the mean (SD) negotiated facility fee for a prostatectomy at compliant hospitals ($9594 [$256]) was $1677 higher than that of noncompliant hospitals ($7917 [$367]; *P* = .001).

## Discussion

In this cross-sectional study, hospitals serving more disadvantaged populations were less compliant with the Hospital Price Transparency final rule over time between Q1 2022 and Q3 2023. Given that racial minority and low-income groups are more likely to experience health care–related financial stress, our findings reveal a harmful paradox: less transparency information available for disadvantaged patients, who have the greatest need to make financially informed decisions.

Our findings also challenge a common assumption that hospitals may be noncompliant to avoid disclosing high prices that could harm brand or market position.^6^ Broad-reaching, payer-disclosed pricing data revealed that compliant hospitals often had higher facility fees compared with noncompliant hospitals.

Study limitations include that we were unable to ascertain certain granular data, such as hospital payer mix or patient out-of-pocket costs, which vary across plan designs. Additionally, prices for these 10 oncologic procedures may not be generalizable to other services. Nevertheless, our findings suggest that CMS should consider policies to avoid regressive financial penalties^2^ on hospitals caring for disadvantaged populations. Instead, policies should promote capacity to help patients make financially informed care decisions with increased technical support or targeted funding for information technology modernization.

## Notes

Supplement 1.
eMethods.
**eFigure.** Hospitals Included in Analysis of Compliance Status and Private Payer-Negotiated Prices of Oncologic Surgeries
eReferences.

Supplement 2.
Data Sharing Statement
