# Supplementary material for: Price Transparency Compliance Among Hospitals Caring for Disadvantaged Populations
Source: JAMA Netw Open. 2026 Apr 10;9(4):e266312. doi: 10.1001/jamanetworkopen.2026.6312 (PMC13069457; doi:10.1001/jamanetworkopen.2026.6312)
Supplement: Supplement 1. — eMethods. eFigure. Hospitals Included in Analysis of Compliance Status and Private Payer-Negotiated Prices of Oncologic Surgeries eReferences. [file jamanetwopen-e266312-s001.pdf]

Supplemental Online Content

Hao D, Rathi VK, Ross JS, Sethi RKV, Xiao R. Price transparency compliance among hospitals caring for disadvantaged populations. *JAMA Netw Open*. 2026;9(4):e266312. doi:10.1001/jamanetworkopen.2026.6312

eMethods.
eFigure. Hospitals Included in Analysis of Compliance Status and Private Payer-Negotiated Prices of Oncologic Surgeries
eReferences.

This supplemental material has been provided by the authors to give readers additional information about their work.

© 2026 Hao D et al. *JAMA Network Open*.

eMethods

Hospital Characteristics

We used several data sources to characterize hospitals in our cohort. We used Turquoise to obtain quarterly compliance ratings for hospitals on a scale of 1 to 5.[1] This rating grades the machine-readable file (MRF) as posted by the hospital on over 60 pieces of information.[2] A score of 1 indicates complete absence of a MRF or shoppable service tool; a score of 5 indicates complete compliance with a MRF with all negotiated rates and a shoppable service tool.[1] More specifically, star ratings were graded on the following scale:

- 5 stars: MRF with negotiated rates (cash and commercial prices)
- 4 stars: MRF is present but missing one key element (codes, key services the provider is known to offer, or cash rates)
- 3 stars: MRF is present but with multiple key elements listed above are missing, but commercial and cash rates are still listed
- 2 stars: MRF is present but with only cash, Medicare, or Medicaid rates without commercial rates
- 1 stars: No MRF available

In this study, we defined a hospital as being "compliant" (yes/no) if it had a rating of either 4 or 5 stars, with either score indicating that the MRF was high-quality, usable, and covered most items/services. Scores of 3 or lower were considered noncompliant.

We characterized the extent to which hospitals care for disadvantaged patients using two types of metrics: Lown Index ratings and CMS Disproportionate Share Hospital (DSH) patient percent value. The Lown Institute created the index to assess more than 3,000 US hospitals on

© 2026 Hao D et al. *JAMA Network Open*.

social responsibility metrics such as health equity, value of care, and patient outcomes.[3,4] We used the Lown Index to measure hospital (1) racial inclusivity and (2) income inclusivity. These measures are defined on a scale of 1 to 5 stars, with 1 star representing the lowest level.[5] Hospitals have higher scores if they have higher patient counts from ZIP codes with greater proportions of non-white patients and lower incomes compared to their “community area,” a radius defined as the distance from which 90% of the hospital's Medicare patients travel. We categorized hospital inclusivity in three-tiers: low (1 or 2 stars), medium (3 stars), or high (4 or 5 stars).

We used the FY 2024 IPPS Correcting Amendment to determine DSH patient percent value.[6] The DSH percent value for each hospital is the sum of Medicare Supplemental Security Income (SSI) and Medicaid fractions for inpatient days. We interpreted the DSH percent value as a surrogate measure for the percentage of low-income patients served. We sorted hospitals into 3 levels corresponding to their DSH percent value on a tertile scale, with 1 representing the lowest DSH percent value.

Relationship Between Hospital Compliance And Pricing

We included the top 10 oncologic surgeries performed in hospitals by 2022 Medicare part B summary claims with a mean Medicare reimbursement greater than $500. We extracted all negotiated facility fees for associated CPT codes without modifiers as disclosed by payers using Turquoise’s Rate Sense database which includes negotiated pricing data from all large national payers and the top 5 payers in every state.[7] For each included procedure, we calculated a median negotiated price at each included hospital and adjusted for hospital wage index to account for geographic differences in cost of care delivery; we then summarized median prices across

© 2026 Hao D et al. *JAMA Network Open*.

hospitals with means with standard errors and compared prices between compliant and noncompliant hospitals using student's t-tests.

© 2026 Hao D et al. *JAMA Network Open*.

eFigure. Hospitals Included in Analysis of Compliance Status and Private Payer-Negotiated Prices of Oncologic Surgeries

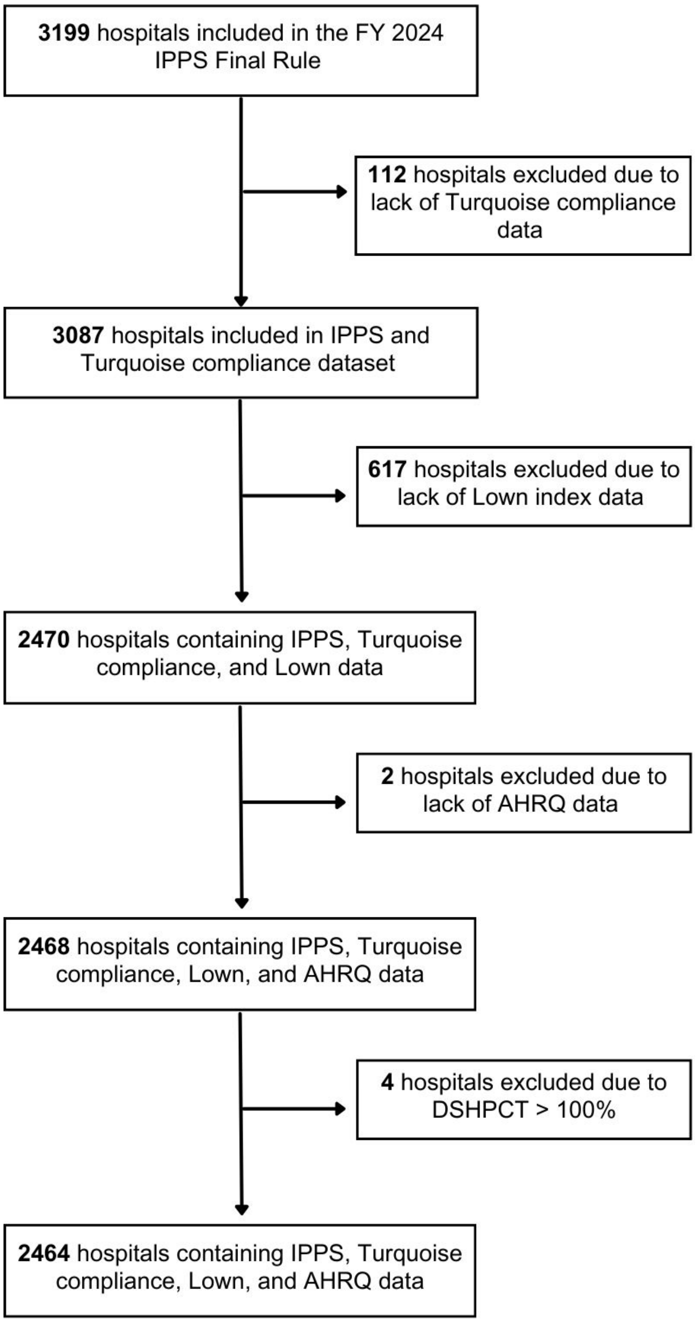

Hospital cohort based on most recent publicly available IPPS Correcting Amendment (2024), Turquoise compliance data, Lown Index data, and AHRQ data. DSHPCT, disproportionate share hospital percent.

© 2026 Hao D et al. *JAMA Network Open*.

eReferences

1. Where we get our data and how it's scored | Turquoise Health. Accessed January 23, 2026. https://turquoise.health/mrf_transparency_score/disclaimers

2. Here's how we calculate our scorecard attributes | Turquoise Health. Accessed August 1, 2023. https://turquoise.health/mrf_transparency_score/scorecard-attributes

3. Lenzer J. "Best" hospitals in US get poor marks in new rating system. *BMJ*. 2020;370:m2725. doi:10.1136/bmj.m2725

4. Blagev DP, Barton N, Grissom CK, McKee KE, Harrison AM. On the Journey Toward Health Equity: Data, Culture Change, and the First Step. *NEJM Catal*. 2021;2(7). doi:10.1056/CAT.21.0118

5. Jung J, Carlin C, Feldman R, Tran L. Implementation of resource use measures in Medicare Advantage. *Health Serv Res*. 2022;57(4):957-962. doi:10.1111/1475-6773.13970

6. FY 2024 IPPS Final Rule Home Page | CMS. Accessed January 24, 2026. https://www.cms.gov/medicare/payment/prospective-payment-systems/acute-inpatient-pps/fy-2024-ipps-final-rule-home-page

7. Rate Sense | Turquoise Health. Accessed July 3, 2023. https://turquoise.health/products/rate_sense

© 2026 Hao D et al. *JAMA Network Open*.
