# Supplementary material for: Price Transparency Compliance Among Hospitals Caring for Disadvantaged Populations
Source: JAMA Netw Open. 2026 Apr 10;9(4):e266312. doi: 10.1001/jamanetworkopen.2026.6312 (PMC13069457; doi:10.1001/jamanetworkopen.2026.6312)
Supplement: Supplement 2. — Data Sharing Statement [file jamanetwopen-e266312-s002.pdf]

Data Sharing Statement

Hao. Price Transparency Compliance Among Hospitals Caring for Disadvantaged Populations. *JAMA Netw Open*. Published online April 10, 2026. doi:10.1001/jamanetworkopen.2026.6312

Data
Data available: Yes
Data types: Data (not involving human participants)
How to access data: Pricing data without any patient data can be made available.
When available: With publication

Supporting Documents
Document types: None

Additional Information
Who can access the data: Anyone requesting data can have access.
Types of analyses: Any purpose.
Mechanisms of data availability: With investigator support.
Any additional restrictions: The data are from Turquoise Health, so within the limitations set by the company for further distribution.
